# Fibrinogen-related proteins in ixodid ticks

**DOI:** 10.1186/1756-3305-4-127

**Published:** 2011-07-05

**Authors:** Jan Sterba, Jarmila Dupejova, Miroslav Fiser, Marie Vancova, Libor Grubhoffer

**Affiliations:** 1 Faculty of Sciences, University of South Bohemia, Branisovska 31, 37005 Ceske Budejovice, Czech Republic; 2 Institute of Parasitology, Biology Centre of the Academy of Sciences of the Czech Republic, Branisovska 31, 37005 Ceske Budejovice, Czech Republic

## Abstract

**Background:**

Fibrinogen-related proteins with lectin activity are believed to be part of the tick innate immune system. Several fibrinogen-related proteins have been described and characterised mainly on the basis of their cDNA sequences while direct biochemical evidence is missing. One of them, the haemolymph lectin Dorin M from the tick *Ornithodoros moubata *was isolated and characterised in more depth.

**Results:**

Several fibrinogen-related proteins were detected in the haemolymph of ixodid ticks *Dermacentor marginatus*, *Rhipicephalus appendiculatus*, *R. pulchellus*, and *R. sanguineus*. These proteins were recognised by sera directed against the tick lectin Dorin M and the haemagglutination activity of the ticks *R. appendiculatus *and *D. marginatus*. Cross-reactivity of the identified proteins with antibodies against the fibrinogen domain of the human ficolin was also shown. The carbohydrate-binding ability of tick haemolymph was confirmed by haemagglutination activity assays, and this activity was shown to be inhibited by neuraminic acid and sialylated glycoproteins as well as by N-acetylated hexosamines. The fibrinogen-related proteins were shown to be glycosylated and they were localised in salivary glands, midguts, and haemocytes of *D. marginatus*. Hemelipoglycoprotein was also recognised by sera directed against the fibrinogen-related proteins in all three *Rhipicephalus *species as well as in *D. marginatus*. However, this protein does not contain the fibrinogen domain and thus, the binding possibly results from the structure similarity between hemelipoglycoprotein and the fibrinogen domain.

**Conclusions:**

The presence of fibrinogen-related proteins was shown in the haemolymph of four tick species in high abundance. Reactivity of antibodies directed against ficolin or fibrinogen-related proteins with proteins which do not contain the fibrinogen domain points out the importance of sequence analysis of the identified proteins in further studies. Previously observed expression of fibrinogen-related proteins in haemocytes together with the results of this study suggest involvement of fibrinogen-related proteins in tick immunity processes. Thus, they have potential as targets for anti-tick vaccines and as antimicrobial proteins in pharmacology. Research on fibrinogen-related proteins could reveal further details of tick innate immunity processes.

## Background

Together with mosquitoes, ticks are the primary vectors of a broad-range of dangerous human and animal pathogens. Some of the well-known infections transmitted by hard ticks (Ixodidae) are Rocky Mountain spotted fever, ehrlichiosis, tick-borne encephalitis, Lyme borreliosis, theileriosis or babesiosis while soft ticks (Argasidae) are vectors of African swine fever and tick-borne relapsing fever [1,2].

Tick haemolymph is a complex fluid composed of plasma and haemocytes. It serves several functions such as transportation of nutrients but it contains also the components of the immune system and helps the tick to fight injuries. Until now, haeme-storage proteins [3,4], defensins [5-7], a tick haemolymph lectin Dorin M from *Ornithodoros moubata *[8,9], and several others have been described.

Tick defence against pathogenic microorganisms is based on recognition of pathogen-associated molecular patterns (PAMPs) such as lipopolysaccharides or peptidoglycans with lectins [10]. Invertebrate/arthropod lectins are believed to be functional analogues of immunoglobulins due to their specific binding to surface carbohydrate structures of pathogens [11]. In arthropods, fibrinogen-related proteins (FRePs) are described as humoral factors of the innate immune system with the ability to recognise PAMPs. FRePs are molecules containing fibrinogen-related domain in the C-terminus; some of these domains exhibit carbohydrate-binding activity [12,13]. FRePs from *Tachypleus tridentatus *(Tachylectin 5A and 5B) with high-sequence homology to human ficolins selectively bind terminal *N*-acetyl group of sugars [14] and this lectin-activity is important in the innate immunity processes [12].

Lectins/FRePs have been found in several ticks [8,15-17] in the haemolymph, the midgut, and salivary glands (SGs) [reviewed in [11]]. Generally, tick lectins display affinity towards sialic acid [8] and *N*-acetyl-D-glucosamine (GlcNAc) [15] as well as galactose [17]. Several FReP-encoding sequences were also described to date: Ixoderin A, Ixoderin B, Ixoderin A-like (*Ixoderin ricinus*), Dorin M, and OMFREP (*O. moubata*) [18]. The OMFREP mRNA is detected in haemocytes and SGs. Ixoderin A is expressed in haemocytes, SGs, and the midgut; Ixoderin B is expressed mainly in SGs and weakly in haemocytes [18]. Sequence similarity of the identified tick lectins with Tachylectins suggests their involvement in innate immunity [9]. Dorin M is still the only purified tick lectin. It is a 640 kDa homomer composed of subunits with 37 kDa [8] and exhibits haemagglutination activity, which is inhibited by *N*-acetylated hexoses, *N*-acetyl neuraminic acid, and sialylated glycoproteins [8,9]. The protein is glycosylated and as such it is recognised by several lectins specific for high-mannose and complex glycans [8]. Three *N*-glycosylated sites described in the protein bear two high-mannose glycans with up to nine mannose residues and a core-fucosylated paucimannosic structure [19].

In *I. ricinus*, 85 kDa lectin was partially characterised with specificity for *N*-acetyl neuraminic acid, *N*-acetyl glucosamine, and D-galactose; however, its sequence is not known [15]. The protein was localised to tick haemocytes and other tissues [16]. Haemagglutination activity of the *Rhipicephalus appendiculatus *haemolymph, the midgut, and SGs have been also characterised. This activity was higher in blood-fed ticks compared with unfed ticks [20].

Furthermore, hemelipoglycoprotein was recognised by serum directed against the Dorin M and against the haemagglutination activity of the *D. marginatus *haemolymph [4]. However, the protein does not show similarity to fibrinogen or fibrinogen-related proteins [3]. Structural similarity of the protein to the fibrinogen domain was therefore suggested [4].

Herein, we describe reactivity of the haemolymph of several ticks with sera against the previously identified tick lectin Dorin M and against haemagglutination activity of the tick haemolymph as well as with antibodies recognising the fibrinogen domain of the human ficolin. The observed fibrinogen-related proteins were further characterised in regards of their glycosylation and they were localised in tissues of the tick *D. marginatus*.

## Results

### Tick haemolymph haemagglutination

Haemagglutination activity (HA) analyses of tick haemolymph samples isolated from *Dermacentor marginatus*, *Rhipicephalus appendiculatus*, *R. pulchellus*, and *R. sanguineus *were performed using 2% suspension of rabbit erythrocytes. In all studied ticks, the haemolymph exhibited haemagglutination activity; HA titre was 512 for *D. marginatus *haemolymph and 192 in the case of the three *Rhipicephalus *haemolymphs (not shown).

Next, haemagglutination inhibition was studied using monosaccharides and glycoproteins (see Table 1). The most potent inhibitors of HA for each tick species were *N*-acetyl neuraminic acid (NeuAc) and sialylated glycoproteins. Moreover, *N*-acetylated hexosamines showed inhibition of the agglutination. However, differences in HA inhibition were detected among tick species and the highest inhibition was observed in *R. sanguineus*.

**Table 1:** Haemagglutination inhibition assays on tick haemolymph.

Saccharide/glycoprotein	**D. m**.	**R. a**.	**R. p**.	**R.s**.
NeuAc	0.0625	0.0312	0.0312	0.0312

ManNAc	0.125	0.0624	0.5	0.0312

GalNAc	0.125	0.5	0.5	0.0625

GlcNAc	0.125	0.125	-	0.0625

L-fucose	-	0.5	-	0.25

BSM	0.00244	0.0312	0.0312	0.0312

PSM	5	0.25	0.5	0.25

fetuin	1.67	0.5	-	0.0625

Polysialic acid	0.208	nd	nd	nd

asialofetuin	3.33	nd	nd	nd

The following saccharides and glycoproteins were used: *N*-acetyl neuraminic (sialic) acid (NeuAc), N-acetyl mannosamine (ManNac), *N*-acetyl galactosamine (GalNAc), *N*-acetyl glucosamine (GlcNAc), fucose, polysialic acid, bovine submaxillary mucine (BSM), porcine stomach mucine (PSM), fetuin, asialofetuin. Except for asialofetuin, all glycoproteins are sialylated. Concentration inhibiting HA is depicted in mol/l for monosaccharides and mg/ml for polysialic acid and glycoproteins. nd - not determined.

We studied haemagglutination activity in ticks *D. marginatus *(D.m.), *R. appendiculatus *(R.a.), *R. pulchellus *(R.p.), *R. sanguineus *(R.s.).

### Reactivity of the tick haemolymph with antibodies to putative FRePs

We used different rabbit/mouse sera directed against potential FRePs: the tick lectin Dorin M (closely related to fibrinogen domain of ficolins); haemolymph lectin molecules with HA activity of ticks *D. marginatus *(anti-(DM)HA) and *R. appendiculatus *(anti-(RA)HA); and immune sera raised against putative *D. marginatus *FReP proteins from this study with molecular weights of 36 kDa (anti-DMF1) and 290 kDa (anti-DMF3). Moreover, we tested antibodies directed against *I. ricinus *lectin (85 kDa) [16] with results corresponding to findings obtained using the immune sera against the potential FRePs (data not shown).

**Figure 1:**
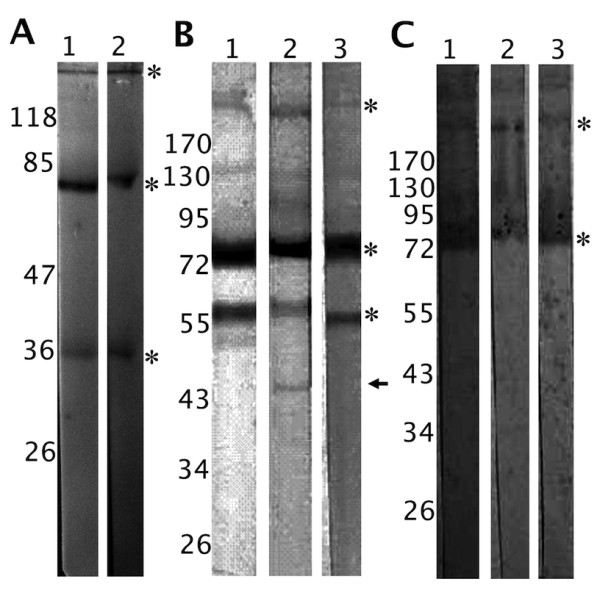
**Immunodetection of the putative fibrinogen-related proteins in tick haemolymph**. **1A **- Electrophoretically separated and electroblotted non-reduced *D. marginatus *haemolymph proteins immunostained using mouse anti-(DM)HA serum (lane 1) and rabbit anti-Dorin M serum (lane 2). Four proteins were detected with molecular weights of approximately 36, 79/80, and 290 kDa (marked with asterisks; the 79/80 kDa double-band is marked by one asterisk). **1B **- Electrophoretically separated and electroblotted non-reduced *R. appendiculatus *(lane 1), *R. pulchellus *(lane 2), and *R. sanguineus *(lane 3) haemolymph proteins immunostained using mouse anti-(RA)HA serum. In each sample, three proteins were detected with size of 58, 75, and 185 kDa (asterisks). Furthermore, a protein band with molecular weight of 45 kDa was observed in *R. pulchellus *(arrow). **1C **- Electrophoretically separated and electroblotted non-reduced *R. appendiculatus *(lane 1), *R. pulchellus *(lane 2), and *R. sanguineus *(lane 3) haemolymph proteins immunostained using rabbit anti-Dorin M serum. Proteins with molecular weight of 75 and 185 kDa were detected, as in the case of anti-(RA)HA serum (asterisks).

When assessing the presence of potential FRePs in the *D. marginatus *haemolymph by immunoblotting, four proteins were identified by both anti-(DM)HA and anti-Dorin M antibodies with molecular weights of approximately 36 kDa, 79/80 kDa, and 290 kDa under non-reducing conditions (Figure 1A). Three proteins were recognised by anti-(RA)HA serum also in *R. appendiculatus*, *R. pulchellus*, and *R. sanguineus *haemolymphs, where the molecular weights of the detected putative FReP proteins were 58 kDa, 75 kDa, and approximately 290 kDa (Figure 1B). Moreover, weak reaction was observed for a 45kDa protein in *R. pulchellus *(Figure 1B, lane 2); however, this protein was not observed in the other *Rhipicephalus *species. The 75 kDa and 290 kDa protein bands were detected also by anti-Dorin M serum (Figure 1C).

### Reactivity of the putative tick FRePs with anti-ficolin antibodies

To confirm the similarity of the identified tick proteins to the fibrinogen domain, we performed immunoblotting of haemolymph proteins from the studied ticks using two commercial antibodies directed against the fibrinogen domain of the human ficolin 1: anti-FCN1 H and anti-FCN S.

**Figure 2:**
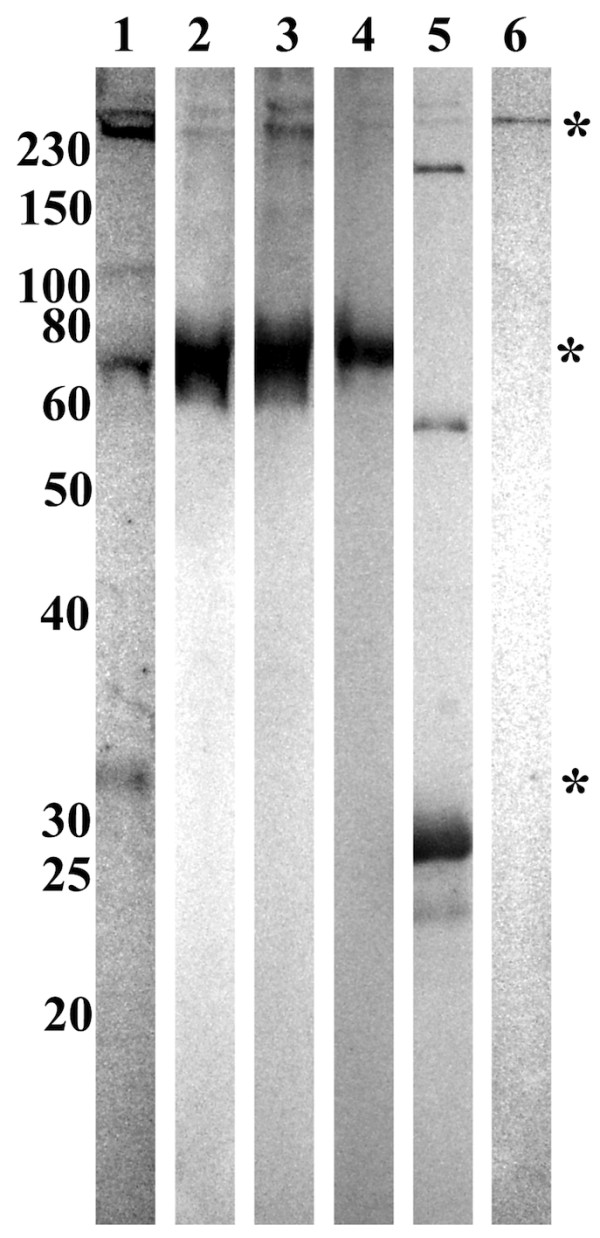
**Immunoblotting of tick haemolymph proteins with anti-ficolin antibodies**. Electrophoretically separated and electroblotted non-reduced haemolymph proteins from *D. marginatus *(lane 1), *R. appendiculatus *(lane 2), *R. pulchellus *(lane 3), and *R. sanguineus *(lane 4) were immunostained using rabbit anti-FCN1 H antibodies. Recombinant human ficolin 1 was used as a control (lane 5). Purified hemelipoglycoprotein from *D. marginatus *haemolymph, which was identified by MS as one of the recognised proteins was used as a control (lane 6). However, this protein does not contain the fibrinogen domain [3]. The same proteins as in Figure 1A were detected in *D. marginatus *haemolymph (36 kDa, 79/80 kDa, and 290 kDa proteins; marked with asterisks; the 79/80 kDa double-band is marked by one asterisk). In *Rhipicephalus *ticks haemolymphs, the 72 kDa and 290 kDa proteins were detected, but not the 55 kDa protein. Additionally, the purified hemelipoglycoprotein from *D. marginatus *was detected by the anti-FCN1 H antibodies. Non-reduced recombinant human ficolin 1 served as a positive control. Antibodies positively reacted with subunits of the protein (approximately 30 kDa) as well as with higher molecular weight complexes (approximately 60 kDa, 180 kDa, 250 kDa, 280 kDa).

All four (36 kDa, 79/80 kDa, and 290 kDa) proteins were recognised in *D. marginatus *haemolymph by the anti-FCN1 H antibodies (Figure 2, lane 1). In the three *Rhipicephalus *species haemolymphs, only the 75 and 290 kDa proteins were recognised (Figure 2, lanes 2, 3, and 4). Furthermore, we performed the immunoblotting on the purified hemelipoglycoprotein from *D. marginatus *[4]; the purified protein was recognised by the anti-FCN1 H antibodies as well (Figure 2, lane 6). Recombinant human ficolin 1 was used as a control (Figure 2, lane 5). Similar results were obtained also using the second antibodies, anti-FCN1 S (data not shown).

### Putative FRePs in tick haemolymph are glycosylated

Positive glycoprotein staining (either non-specific Schiff periodate staining or glycan-specific lectinoblotting) of tick haemolymph proteins revealed abundant glycosylation of proteins. Protein bands corresponding to putative FReP proteins were also positively stained (data not shown). To address the glycosylation of these proteins more specifically, we performed enzymatic deglycosylation reactions of the haemolymphs from *D. marginatus*, *R. appendiculatus*, and *R. sanguineus *ticks. After deglycosylation, putative FRePs were detected using anti-(DM)HA, anti-(RA)HA, anti-DMF1 or anti-DMF3 sera. Thus, changes in molecular weights of the putative FRePs could be detected without the interfering staining of the other haemolymph proteins.

**Figure 3:**
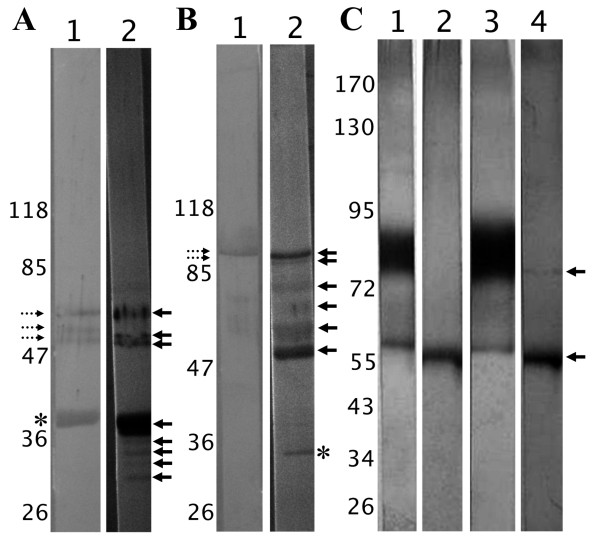
**Deglycosylation of the putative fibrinogen-related proteins in tick haemolymph and their immunodetection**. **3A **- Reduced *D. marginatus *haemolymph proteins (lane 1) were enzymatically deglycosylated (lane 2). The FReP proteins were detected using anti-DMF1 serum, which was raised against the 36 kDa protein (asterisk). After deglycosylation, additional bands appeared with sizes of 31, 33, and 34 kDa (arrows). Cross-reactivity of the serum with 79/80 kDa proteins was observed (bands with molecular weights of 58, 60, and 66 kDa - lane 1, dotted arrows). The molecular weight of these proteins shifted after deglycosylation and three bands were observed at 54, 58, and 63 kDa (lane 2, arrows). **3B **- Reduced *D. marginatus *haemolymph proteins (lane 1) a were enzymatically deglycosylated (lane 2). The FReP proteins were detected using anti-DMF3 serum, which was raised against the 290 kDa protein. This protein is composed of two subunits which have 95 and 100 kDa (lane 1, dotted arrows). After deglycosylation (lane 2), additional bands appeared at 50 and 74 kDa (arrows). We observed cross-reactivity of the serum also in this case, when the same bands were observed for the 79/80 kDa proteins (lane 2, arrows) as in the case of anti-DMF1 serum (see Figure 2A). The protein band at 34 kDa (lane 2, asterisk) is probably a protein cleavage product. **3C **- Reduced *R. appendiculatus *(lanes 1,2) and *R. sanguineus *(lanes 3,4) haemolymph proteins before (lanes 1,3) and after deglycosylattion (lanes 2,4). The FReP proteins were detected using anti-(RA)HA serum. In both tick samples (lanes 1,3), 58 kDa band was observed and a protein smear from 75 to 90 kDa (75 kDa protein and the subunits for 185 kDa protein). The deglycosylation diminished the reactivity of the protein smear and only the band at 58 kDa remained visible (lanes 2,4; arrows).

In all cases, deglycosylation of haemolymph proteins changed the FRePs immunostaining pattern. In *D. marginatus *haemolymph, anti-DMF1 serum stained the 36 kDa protein under reducing conditions. After deglycosylation with *N*-glycosidase F (cleaves off the whole *N*-glycan except for structures containing core α(1,3)-bound fucose), reactive protein bands with molecular weights of 30, 31, 33, 34, and 36 kDa appeared (Figure 3A). The enduring reaction of the 36 kDa band suggests incomplete deglycosylation reaction. The anti-DMF1 serum detected also the 79/80 kDa proteins, which under reducing conditions migrated as three protein bands with molecular weights of 58, 60, and 66 kDa suggesting the presence of non-covalently bound subunits. The molecular weights of these three bands decreased after *N*-glycosidase F treatment and they were observed at 54, 58, and 63 kDa (Figure 3A). Same results with lower intensity were obtained for the 79/80 kDa proteins also using anti-DMF3 serum (Figure 3B).

Anti-DMF3 serum stained two bands at 95 and 100 kDa under reducing conditions suggesting non-covalently bound subunits in the 290 kDa protein. After the *N*-glycosidase F treatment, four bands belonging to the protein were observed with molecular weights of 50, 74, 95, and 100 kDa. While the larger bands suggest incomplete deglycosylation, appearance of the 50 and the 74 kDa bands shows cleavage of the glycan moiety from the subunits of the 290 kDa protein (Figure 3B). Another stained protein at Mw of 34 kDa also appeared on the blot.

The reactivity of anti-(RA)HA serum with deglycosylated proteins from the *Rhipicephalus *ticks haemolymphs markedly decreased (Figure 3C). In haemolymph under reducing conditions, the serum recognised the 58 kDa protein and a smear from 75 to 90 kDa, which we speculate represents the 75 kDa protein as well as the subunits of the 290 kDa protein. After deglycosylation using Endo H enzyme, which cleaves between the two innermost GlcNAcs of the *N*-glycans, only one protein remained reactive with molecular weight of 56 kDa in both *R. appendiculatus *and *R. sanguineus *haemolymphs. In *R. sanguineus*, a slight reaction at 75 kDa was visible, representing possibly the incompletely deglycosylated protein (Figure 3C).

The *N*-glycosidase F treatment on *Rhipicephalus *ticks haemolymph as well as deglycosylation of *D. marginatus *haemolymph using Endo H enzyme resulted in disappearance of the anti-(RA)HA or anti-Dorin M sera staining (data not shown).

### Localisation of FRePs in *D. marginatus *organs

Taking advantage of specific anti-FReP sera, we performed immunolocalisation of these proteins in the midgut, SGs, and haemocytes dissected from the partially fed *D. marginatus*.

**Figure 4:**
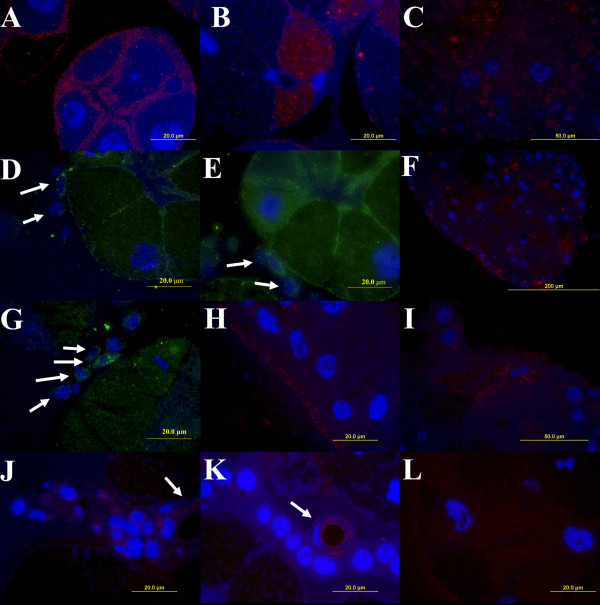
**Immunolocalisation of FRePs in *D. marginatus *organs using fluorescence microscopy**. Thin sections of midgut (C, F, H, I), salivary glands (A, B, J, K, L), and haemocytes attached to salivary glands (D, E, G) from fed female *D. marginatus *were labelled with sera raised against *D. marginatus *FRePs. Anti-DMF1 (A, B, C, D, E), anti-DMF2 (F, G), and anti-DMF3 (H, I, J, K) antibodies were used. Control reaction were carried out, in which anti-DMF sera were omitted (L). FRePs were localised on the surface and inside epithelial cells surrounding secretory granular cells of the acini type II (A, D, E); in granules of secretory cells located in the acini type III (B) and in haemocytes attached to the surface of salivary glands (D, E, G; arrows). Positive labelling reactions were observed in the cytoplasm of epithelium of salivary duct cells (J, K; arrows) and in thin cuticular layer facing to the lumen of the salivary duct (J, K). Anti-DMF sera detected structures on the surface (H) and inside the midgut cells (C, F, I). A, B, C, F, H, I, J, K, and L - Cy3-conjugated secondary antibodies (red); D, E, and G - FITC conjugated secondary antibodies (green). Cell nuclei were counter-stained using DAPI (blue).

In a type III of SG acini, anti-DMF1 serum labelled structures inside the epithelial cells that surround the secretory cells (Figure 4A). In the acinus type II, positive reaction of this serum was detected inside of the secretory granules located in the cytoplasm of cells occurring near the acinar duct (b cells; Figure 4B). We observed anti-DMF1 labelling inside the midgut cells (Figure 4C) and, surprisingly, in perinuclear region of haemocytes attached to SGs (Figures 4D, E). Only haemocytes attached to SGs showed reactivity with anti-DMF1 serum while free circulating haemocytes did not appear to contain FRePs (data not shown).

Anti-DMF2 serum showed the presence of FRePs in the cytoplasm of midgut cells (Figure 4F) and inside of haemocytes attached to SGs (Figure 4G). Again, circulating haemocytes did not appear to contain FRePs (data not shown).

Anti-DMF3 serum localised FRePs in surface structures above midgut cells as seen in the longitudinal section (Figure 4H) and inside of several surface cells of the midgut as evident from the tangential section (Figure 4I). Anti-DMF3 labelling was observed inside the salivary duct in the cytoplasm of epithelium and in the cuticular layer facing to the lumen of the salivary duct (Figures 4J,K).

### Identification of FRePs by mass spectrometry

Coomassie Brilliant Blue-stained protein bands, corresponding to the positively immunostained putative FRePs, were cut off the SDS-PAGE gel, alkylated and reduced, and finally trypsin digested. Next, the tryptic peptides were analysed by mass spectrometry. The 290 kDa proteins from *D. marginatus *and from the three *Rhipicephalus *species were identified as hemelipoglycoprotein from *D. marginatus *(GenBank: ABD83654, Table 2). Identification of the other putative FRePs was not successful. However, examination of the hemelipoglycoprotein amino acid sequence (from *D. variabilis*, [4]) did not show the presence of the fibrinogen domain.

**Table 2:** Summary of the MS identification experiments performed on the 290 kDa proteins from the studied ticks.

	Number of repetitions	Amino Acid sequence coverage
*D. marginatus*	5	10.2%

*R. appendiculatus*	3	13.4%

*R. pulchellus*	3	4.2%

*R. sanguineus*	3	20.8%

Number of MS experiments performed for identification of each of the 290 kDa proteins from the ticks *D. marginatus*, *R. appendiculatus*, *R. pulchellus*, and *R. sanguineus*. All the proteins were identified as hemelipoglycoprotein (*D. variabilis *hemelipoglycoprotein GenBank:ABD83654) and the amino acid sequence coverage is indicated for each protein.

## Discussion

These results provide further information about tick lectins/FRePs. In agreement with our previously published results, we detected haemagglutination activity (HA) in haemolymph of ticks *Dermacentor marginatus*, *Rhipicephalus appendiculatus*, *R. pulchellus*, and *R. sanguineus *[15]. HA was previously detected in *R. appendiculatus *and it was inhibited by GalNAc, xylose, and fructose [21,22]. Our results show *N*-acetylated saccharides and sialic acid together with sialylated glycoproteins as the most potent inhibitors of HA in all studied *Rhipicephalus *species and in *D. marginatus*. These observations suggest involvement of NeuAc and the *N*-acetyl groups in saccharide recognition by tick lectins/FRePs. Previously, we observed similar inhibitory pattern also for *Ixodes ricinus*, *Ornithodoros tartakovskyi*, *O. pappillipes*, and *Argas polonicus *[15].

The haemagglutinins of the tick haemolymph seem to belong to the fibrinogen-related protein family, as was shown in the case of the lectin Dorin M from *O. moubata *which exhibited HA [8]. Haemagglutination is commonly mediated by carbohydrate specific binding and thus, by lectins [23]. Immune sera against the haemagglutination activity are therefore used for detection of unknown lectins [20].

Antigenic similarities among all examined lectins/FRePs were confirmed by cross-reactivity of antibodies directed against these proteins among various ixodid and argasid tick species. We used the immune serum raised against the Dorin M (which was purified in native state from the haemolymph of *O. moubata *[8]) to detect the presence of FReP proteins also in ixodid ticks. Anti-Dorin M serum reacted with four haemolymph proteins in *D. marginatus *(molecular weights of 38, 79/80, and 290 kDa) and with three protein in *Rhipicephalus *ticks (molecular weights of 58, 75, and 290 kDa). An additional protein with size of 45 kDa was detected in *R. pulchellus*. Similarity of epitopes was shown for putative FRePs from *D. marginatus*, where the sera raised against the different FRePs cross-reacted with all proteins. The same proteins were detected using sera raised against the HA of ticks - *D. marginatus *and *R. appendiculatus *(Figures 1A, B) and *I. ricinus *(data not shown). These antibodies should be directed against lectin-like molecules present in the haemolymph [20]. The same staining patterns obtained using these sera and the anti-Dorin M serum again suggests close relatedness of FReP proteins and the fact that they belong to lectins.

Finally, commercial antibodies directed against the fibrinogen domain of ficolin reacted with the identified putative tick FRePs (Figure 2). Except the biggest recognised protein in each species, hemelipoglycoprotein, none of the proteins was identified by mass spectrometry. Most probably this results from the missing information on gene sequences from almost all tick species; the only known tick genome is that of *I. scapularis*. However, a Dorin M-like protein was identified in cDNA libraries from *D. marginatus *using degenerate primers specific for the fibrinogen domain of Dorin M and other FRePs (GenBank:ACI22373) [24]. As *D. marginatus *hemelipoglycoprotein does not contain the fibrinogen domain, we conclude, that this protein could be one of the smaller putative FRePs identified in this study in *D. marginatus *(36 kDa or 79/80 kDa proteins).

The largest of these proteins in each case were identified as hemelipoglycoprotein (HLGP). The protein was recognised by sera directed against the FReP proteins, HA of tick haemolymphs as well as against the fibrinogen domain itself. However, the protein does not contain the fibrinogen domain and therefore, the protein is not related to FRePs [3]. One of the explanation, could be epitope-similarity of the protein to the fibrinogen domain. Structural rather than sequence-similarity to other fibrinogen or other lectins is implicated also by saccharide-binding by HLGP [4]. However, reactivity of anti-fibrinogen antibodies with HLGP show, that the recognition of protein by anti-FReP antibodies does not necessarily mean the presence of the fibrinogen domain in the recognised protein.

The putative FRePs detected in this study seem to be glycosylated. Glycosylation is the common feature of lectins/glycan-binding proteins [25]. Decrease in molecular weights of the detected proteins was observed after enzymatic deglycosylation in the range of several Daltons, which corresponds to the low-mass arthropod/tick glycans [[4,19,26], unpublished results]. The presented results for hemelipoglycoprotein also correspond to our previous findings [4]. In *D. marginatus*, a small molecular weight protein was detected after deglycosylation (34 kDa) using anti-DMF3 serum. However, the molecular weight difference is too big to be related to the glycosylation and we assume this band to represent a protease cleavage product of the 290 kDa protein.

Further, the newly detected putative FRePs and hemelipoglycoprotein were immunolocalised in *D. marginatus *tissues. One of the FReP-specific sera labelled granules of secretory SGs acini type II suggesting secretion of the proteins to the host. The detection of FRePs in surface parts of acini type III and midgut and especially in haemocytes associated with SGs may indicate their putative innate immune functions. Surprisingly, we were not able to detect FReP proteins in freely circulating haemocytes. Tick FRePs/lectins as well as hemelipoglycoprotein are expressed in same organs as shown by detection of the proteins or their mRNA [4,11,18].

Involvement of carbohydrate-binding proteins in the tick immune reaction was shown previously in the case of *Theilleria parva *infection of *R. appendiculatus *[27]. Thus, the detection of new members of fibrinogen-related protein family in the *Dermacentor *and *Rhipicephalus *ticks, and their further characterisation can bring new information about the tick innate immunity processes and open new ways in struggle with ticks and tick-borne diseases. Hemelipoglycoprotein, which does not contain the fibrinogen domain but seems to share structural features with FRePs was shown to bind saccharides as well [4]. In fish, vitellogenin (closely related to hemelipoglycoprotein of ticks) was shown to be directly involved in immune reaction and in recognition of carbohydrate moieties on the surface of invading bacteria [28].

## Conclusions

Fibrinogen-related proteins with lectin activities are present in all studied tick species. These proteins involved in recognition of NeuAc and the *N*-acetyl groups are expressed in midgut, salivary glands as well as haemocytes attached to the salivary glands. Information about specific activities of these exemplary molecules could reveal much information on tick innate immunity processes and help in future design of anti-tick vaccines. Furthermore, cross-reactivity of antibodies against the FRePs and the fibrinogen domain itself with proteins which do not contain the fibrinogen domain point out the need for sequence analysis of proteins identified by such antibodies.

## Methods

### Ticks

Partially-fed females of ticks *Dermacentor marginatus*, *Rhipicephalus appendiculatus*, *R. pulchellus*, and *R. sanguineus *were obtained from the tick rearing facility of the Institute of Parasitology, Biology Centre of the Academy of Sciences of the Czech Republic in České Budĕjovice. Females were allowed to feed on guinea pigs for 6 days.

### Haemolymph and tissue preparation

Haemolymph was collected after cutting off a part of anterior leg of partially-fed female by fine scissors. The haemolymph from 8 to 10 females (corresponding to 10-15 μl) was collected into 50 μl PBS containing protease inhibitors (Pierce). The solution was centrifuged at 4°C for 10 minutes at 100 × g to pellet the haemocytes. The resulting supernatant was then clarified at 23000 × g for 20 minutes and both the plasma (further referred to as 'haemolymph') and haemocyte fractions were stored at -20°C. Haemolymph was prepared for ticks *D. marginatus R. appendiculatus*, *R. pulchellus*, and *R. sanguineus*.

Midgut and salivary glands were dissected from partially-fed females of the tick *D. marginatus*, thoroughly washed in PBS to remove possible contamination from gut-content, and put into 0.9% NaCl at 4°C before their processing for the fluorescence microscopy analysis.

### Haemagglutination activity (HA) and HA inhibition

Haemagglutination activity assays were performed as described earlier [20] using 2% suspension of rabbit erythrocytes in 0.15 M NaCl for 1 hour at room temperature. Two-fold dilution of haemolymph was prepared in U-type bottom microtitration plates. Haemagglutination buffer (50 mM Tris-HCl, 0.15 M NaCl, 20 mM CaCl2, pH 7.0) was used for the experiments. Reciprocal value of the last dilution of the highest sample dilution still causing visible agglutination was used as the titre of HA and the amount of haemagglutinins in this well is defined as 1 HA unit.

HA inhibition was performed in serial dilution of saccharides and glycoproteins.. Haemolymph diluted to contain 1.5 HA unit and 2% erythrocytes suspension were added. The 50% inhibitory concentration was determined after 1 hour at room temperature as the lowest inhibitor concentration that completely inhibits the binding activity of 1.5 HA unit.

### Commercial antibodies and polyclonal serum preparation

Two commercial antibodies produced in rabbits were used for confirmation of epitope similarities of the studied proteins with ficolin. The first, anti-FCN1 H (HPA001295, Sigma-Aldrich), recognise amino acids 199 to 307 from the human ficolin 1 while the second, anti-FCN1 S (SAB2100804, Sigma-Aldrich), is specific for the region 180-229 of the protein. Thus, both antibodies are specific for the fibrinogen domain of the ficolin, which comprises amino acids 115 to 325.

We have described the preparation of anti-haemagglutination activity (anti-HA) serum elsewhere [20]. Briefly, mouse erythrocytes were incubated with *D. marginatus *(serum referred to as 'anti-(DM)HA') or *R. appendiculatus *(serum referred to as 'anti-(RA)HA') haemolymph, washed, mixed with Freund's adjuvant in a 1:1 ratio and injected into mice. Immunisation was repeated 4 × every 14 days. Blood was collected 14 days after the last immunisation. Sera were supplemented with glycerol (1:1), aliquoted and stored at -20°C.

Polyclonal immune sera against the *D. marginatus *FRePs were prepared using the respective proteins cut out the SDS-PAGE gel. Anti-DMF1 serum was raised against the 36 kDa protein, anti-DMF2 against the 79/80 kDa double-band, and anti-DMF3 against the 290 kDa protein. Each protein band was cut out and homogenised with 60 μl PBS (1×). Freund's adjuvant was added in a 1:1 ratio and 80 μl of this solution was subcutaneously injected to BALB/c mice. Immunisation was repeated 4 × every 14 days. Blood sera were collected 14 days after the last immunisation. Sera were supplemented with glycerol (1:1), aliquoted and stored at -20°C.

Mice were handled according to internal rules of the Institute of Parasitology, BC ASCR, Ceske Budejovice and the Animal Act of the Parliament of the Czech Republic.

### SDS-PAGE and immunoblotting

For SDS-PAGE, haemolymph samples were diluted 1:5 in physiological buffer, mixed with loading buffer (Fermentas) and heated for 5 min at 95°C. SDS-PAGE [29] was performed on 4-17.5% gradient gels. Gels were stained with PageBlue Protein Staining Solution containing Coomassie Brilliant Blue (Fermentas).

The electrophoretically separated proteins were transferred to the PVDF membrane according to [30] for 1 hour at 20 V. The PVDF membrane was washed in PBS (14 mM NaCl, 0.15 mM KH2PO4, 1.8 mM Na2HPO4, 0.27 mM KCl, pH 7.2), cut into strips, and incubated for 1 hour in 5% skim powdered milk in PBS. Strips were then incubated for 1 hour in mouse the appropriate antibodies (see above), washed with PBS-Tween 20 (0.05% Tween 20 in PBS) and incubated with goat anti-mouse/anti-rabbit antibody conjugated with alkaline phosphatase (AP, VectorLabs) in 5% milk. After incubation, strips were washed with PBS-Tween 20 and PBS. Reaction was developed in AP staining solution (VectorLabs) and after the development of sufficient signal was stopped by washing the strips several times in distilled water.

### Enzymatic deglycosylation

Haemolymph samples were deglycosylated using glycosidases Endo H (New England Biolabs) or N-glycosidase F (New England Biolabs) in 50 mM sodium phosphate buffer, pH 7.4, overnight at 37°C, according to the instructions of the manufacturer. Denaturation of proteins using SDS and DTT at 95°C for 10 minutes was performed prior the deglycosylation. Deglycosylation reactions were carried out in duplicates.

### Fluorescence microscopy

SGs and midguts were fixed in 4% formaldehyde/0.1% glutaraldehyde in 0.1 M phosphate buffer, pH 7.4 (PB) for 2 hours at 4°C. Pieces of tissues were embedded in 10% gelatine at 37°C, rinsed in PB at 4°C and dehydrated with a gradient series of ethanol (30% at 0°C, 50%-100% at -15°C for 1 h) in the EM AFS freeze substitution device (Leica). The specimens were gradually infiltrated with 25%, 50%, 75%, and 100% resin LR White (Polysciences) with 0.05 g benzoyl methylether/10 g resin at -15°C. The specimens were embedded to BEEM capsules (Polysciences) and polymerised at -15°C for 24 hours under UV radiation.

After polymerisation, the semithin sections were cut using the ultramicrotome EM UC6 (Leica) and dried on microscopic slides.

The sections were blocked in Tris-buffered saline, pH 7.4 (TBS) with 0.05% Tween-20 containing 3% BSA for 3 hours and incubated with 1:20 anti-DMF1 or anti-DMF2 or anti-DMF3 sera overnight at 4°C. After washing in TBS, the sections were incubated with anti-mouse secondary antibody conjugated with FITC (Sigma-Aldrich) or Cy3 (Jackson Immunoresearch) for 3 hours at ambient temperature, washed thoroughly, and the cell nuclei were stained in 1 μl/ml DAPI (Sigma-Aldrich) solution for 15 minutes at ambient temperature. After washing, the sections were mounted in a solution composed of 2.5% DABCO/95% glycerol containing n-propyl galate (15 mg/ml) (Sigma-Aldrich) and examined using a BX51 fluorescence microscope equipped with a DP70 camera (Olympus).

### Mass spectrometry

Putative FRePs were cut out the SDS-PAGE gels and reduced, alkylated, and trypsinised (ROCHE) according the manufacturer's instructions and subjected to LC-MS analysis. Peptides were separated by NanoAcquity UHPLC (Waters) on C18 silica (BEH300 column, Waters) using acetonitrile gradient (5-80%) as a mobile phase and analysed by ESI-QTOF PREMIER mass spectrometer (Waters). The obtained data were compared to *Acari *non-redundant and Swiss-Prot databases using ProteinLynxGlobalServer software (Waters) under strict criteria.

## Competing interests

The authors declare that they have no competing interests.

## Authors' contributions

JD, MF, JS conducted electrophoreses, blotting experiments, and HA assay, JD and JS participated in anti-DMF and anti-tick HA sera preparation, MV performed tick tissue preparation and together with JD performed the FM analysis, JS prepared the manuscript, and LG co-ordinated the experiments. All authors participated in the design of experiments and they approved the final manuscript.
